# Fibrotic remodeling and stromal reprogramming in osteoarthritis: mechanisms, mechanobiology, and emerging therapeutic perspectives

**DOI:** 10.3389/fimmu.2026.1901489

**Published:** 2026-10-08

**Authors:** Yanbing Liang, Yuxiao Xiang, Weijia Li, Juanli Zhang, Jianxiang Shi

**Affiliations:** 1 Nanyang Central Hospital, Nanyang, Henan, China; 2 School of Osteopathy, Henan University of Chinese Medicine, Zhengzhou, Henan, China; 3 National Medical Products Administration (NMPA) Key Laboratory for Quality Control of In Vitro Diagnostics, Henan Medical Products Administration, Zhengzhou, Henan, China; 4 State Key Laboratory of Metabolic Dysregulation & Prevention and Treatment of Esophageal Cancer, Henan Key Laboratory of Health Medicine and Liver Disease Prevention & Treatment, Henan Institute of Medical and Pharmaceutical Sciences, Zhengzhou University, Zhengzhou, Henan, China

**Keywords:** fibroblast-to-myofibroblast transition, fibrosis, inflammation, mechanotransduction, osteoarthritis, precision medicine, stromal reprogramming, synovial fibrosis

## Abstract

Osteoarthritis (OA) has traditionally been regarded as a degenerative joint disorder characterized primarily by cartilage erosion. However, growing evidence suggests that OA is a disorder of maladaptive whole-joint remodeling in which fibrotic remodeling represents an important yet underappreciated contributor to disease progression. Fibrotic remodeling is best documented in the synovium and joint capsule, with related stromal and extracellular matrix (ECM) changes also occurring in the infrapatellar fat pad and other joint compartments. Recent studies suggest that OA-associated fibrotic remodeling extends beyond passive scar formation and involves persistent stromal reprogramming, chronic inflammatory signaling, and mechanobiological adaptation. In this review, stromal reprogramming is used as a conceptual framework describing persistent alterations in stromal cell state that extend beyond transient fibroblast activation or fibroblast-to-myofibroblast transition (FMT). These alterations may involve sustained transcriptional programs, altered mechanosensitivity, metabolic or epigenetic adaptation, and incomplete restoration of tissue homeostasis. However, direct evidence demonstrating stable long-term stromal reprogramming in human OA remains limited. Advances in single-cell omics, spatial transcriptomics, and systems biology approaches have revealed substantial heterogeneity among stromal and immune cell populations, providing new insights into fibrosis-associated cellular states and potential opportunities for biomarker-guided patient stratification. Nevertheless, whether these states represent stable biological entities, transitional disease stages, or secondary responses to advanced OA remains unresolved. This review focuses on the unresolved transition from adaptive tissue repair to persistent fibrotic remodeling and proposes a conceptual framework linking stromal reprogramming, inflammatory signaling, ECM remodeling, and mechanobiological feedback during OA progression. Throughout, we distinguish direct evidence from human OA and experimental OA models from mechanistic insights derived from rheumatoid arthritis and other fibrotic diseases. We further discuss emerging precision medicine strategies and future opportunities for biomarker-guided and mechanobiology-informed antifibrotic interventions. Although this Review adopts a whole-joint perspective, current mechanistic evidence remains predominantly synovium-centered, highlighting the need for further investigation of fibrotic remodeling across additional joint compartments.

## 1 Introduction

Osteoarthritis (OA) has traditionally been defined as a degenerative joint disease driven by progressive cartilage erosion (1, 2). However, this cartilage-centric view is increasingly insufficient to explain the complexity, persistence, and heterogeneity of disease progression (2, 3). OA is now widely recognized as a disorder of the entire joint organ, involving coordinated pathological changes in cartilage, synovium, subchondral bone, infrapatellar fat pad, joint capsule, ligaments, and periarticular connective tissues (3–5).

Among these processes, fibrotic remodeling has recently gained attention as an important but underappreciated contributor to OA progression (6, 7). Fibrotic remodeling is best documented in the synovium and joint capsule, with related stromal and extracellular matrix (ECM) changes also reported in the infrapatellar fat pad and other joint compartments, contributing to chronic pain, joint stiffness, and functional decline (7–9). Excessive ECM deposition, altered matrix organization, and tissue stiffening contribute to joint dysfunction, pain, and restricted mobility (7–9). Importantly, fibrotic remodeling in OA is increasingly recognized as an active remodeling process rather than simply a passive consequence of tissue injury, although the extent and biological significance of fibrosis may differ among individual joint compartments (7–10).

At the cellular level, OA-associated fibrotic remodeling is closely linked to stromal cell activation and, in persistent disease states, stromal reprogramming, particularly activation of fibroblast-like synoviocytes (FLS), which can acquire myofibroblast-like features characterized by enhanced contractility and ECM production (6, 8, 11, 12). Fibroblast-to-myofibroblast transition (FMT) provides a mechanistic link between inflammatory cues and structural remodeling in the osteoarthritic joint (6, 11). In this review, stromal reprogramming refers to a broader and more persistent alteration in stromal cell state that extends beyond transient fibroblast activation or FMT and may involve sustained transcriptional programs, altered mechanosensitivity, metabolic adaptation, epigenetic stabilization, and incomplete return to tissue homeostasis after withdrawal of the initiating stimulus. At the molecular level, transforming growth factor-β (TGF-β), inflammatory cytokines, and mechanotransduction pathways such as YAP/TAZ form interconnected regulatory networks that influence fibroblast activation, ECM deposition, and tissue stiffening (13–16). Importantly, ECM stiffening can further amplify mechanotransduction signaling, establishing feedback mechanisms that may stabilize profibrotic cellular states (13, 14).

These insights highlight a critical gap in current OA research and therapy. Although substantial efforts have focused on cartilage preservation and symptomatic management, OA-associated fibrotic remodeling remains incompletely characterized and largely untargeted (17, 18). The lack of validated fibrosis-specific biomarkers, together with uncertainty regarding fibrosis-associated OA phenotypes and patient stratification, further constrains the development of effective disease-modifying strategies (19, 20). Several recent reviews have comprehensively summarized synovial fibrosis, FLS phenotypes, FMT, and individual profibrotic pathways in OA (6–8, 11, 21, 22). Unlike these reviews, which primarily focus on synovial fibrosis, fibroblast phenotypes, or individual profibrotic pathways, the present review focuses on the unresolved transition from adaptive tissue repair to persistent stromal reprogramming and fibrotic remodeling, while integrating stromal–immune interactions, mechanobiology, and whole-joint pathology into a unified framework.

To address this question, it is essential to understand why an initially adaptive tissue-repair response fails to resolve and instead progresses toward persistent fibrotic remodeling. Accumulating evidence suggests that fibrotic remodeling in OA extends beyond passive ECM accumulation and involves persistent alterations in stromal cell activation, inflammatory signaling, and mechanobiological adaptation across the joint microenvironment (10, 13, 14). In this context, fibroblasts, immune cells, and ECM components may collectively contribute to chronic wound-like responses characterized by sustained profibrotic signaling and impaired tissue resolution. Although the concept of stromal reprogramming provides a useful framework for interpreting these observations, much of the underlying mechanistic evidence is derived from experimental OA models or other fibrotic diseases and should therefore be interpreted with appropriate caution.

In this review, we synthesize recent advances in the understanding of fibrotic remodeling in OA, with particular emphasis on the interplay among cellular plasticity, inflammatory signaling, mechanobiology, metabolic adaptation, and epigenetic regulation. We propose an integrated framework in which fibrotic remodeling represents an important organizing process that links inflammation, stromal activation, ECM remodeling, and biomechanical alterations during OA progression (**Figure 1**). Throughout the review, we distinguish direct evidence from human OA tissues and experimental OA models from mechanistic insights derived from rheumatoid arthritis, arthrofibrosis, and fibrosis in non-articular organs. We further discuss how emerging precision medicine technologies—including single-cell omics, spatial transcriptomics, and biomarker-guided stratification—may facilitate the development of next-generation antifibrotic therapies.

**Figure 1:**
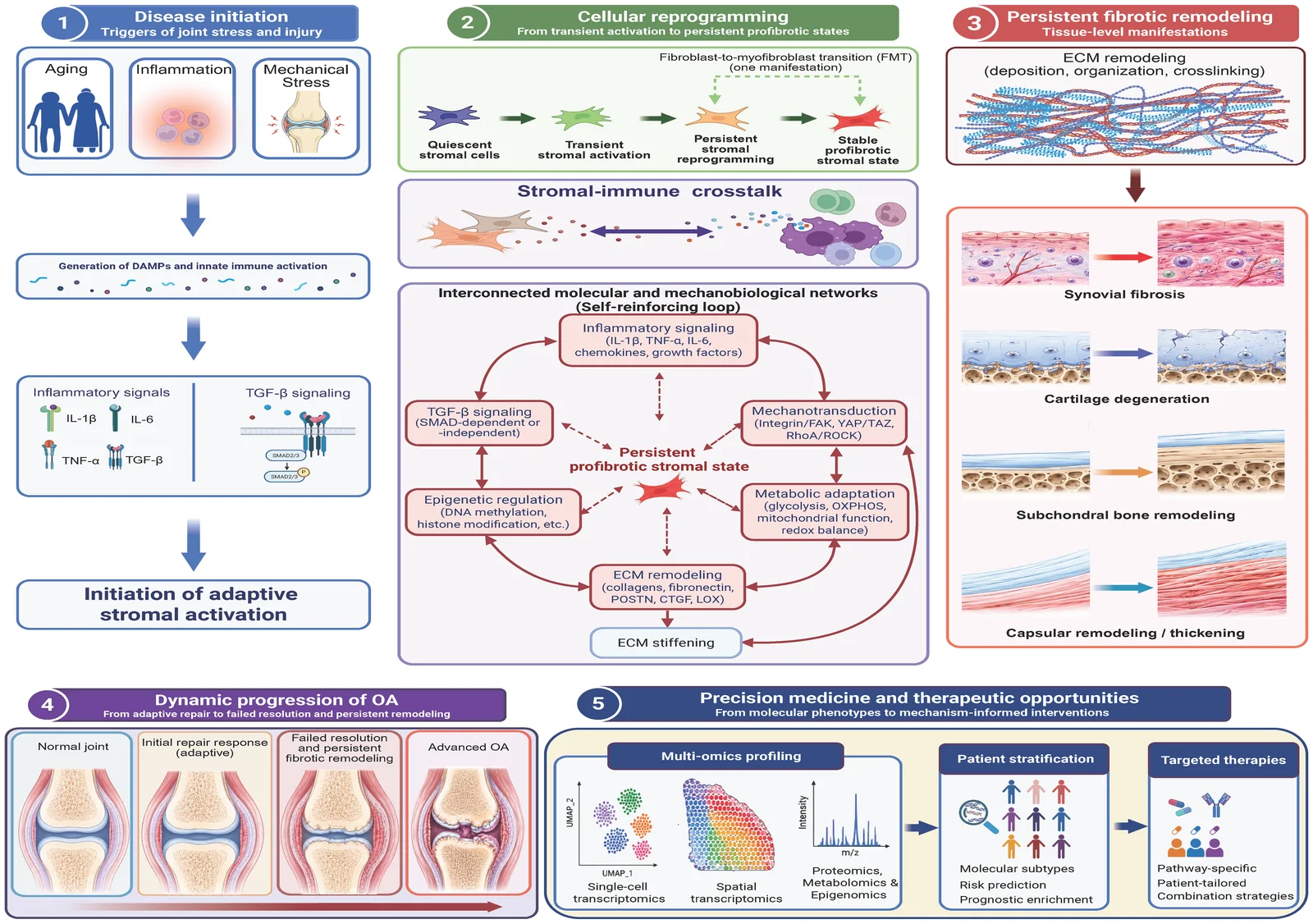
Conceptual framework of whole-joint fibrotic remodeling in osteoarthritis. Aging, inflammation, and mechanical stress initiate joint injury, damage-associated molecular pattern (DAMP) generation, inflammatory signaling, and an initially adaptive stromal response. Failure of resolution may promote persistent stromal reprogramming, with fibroblast-to-myofibroblast transition (FMT) representing one manifestation rather than a synonym of this process. Stromal–immune crosstalk and interconnected inflammatory, TGF-β, metabolic, epigenetic, and mechanobiological pathways may reinforce persistent profibrotic stromal states. Quantitative and qualitative extracellular matrix (ECM) remodeling can promote ECM stiffening, which can enhance mechanotransduction and further matrix production and cellular contractility, thereby establishing a self-reinforcing mechanobiological feedback loop. Persistent fibrotic remodeling is associated with synovial fibrosis and broader whole-joint changes, including cartilage degeneration, subchondral bone remodeling, and capsular remodeling/thickening. Within this framework, OA progression may involve a transition from adaptive repair to failed resolution and persistent remodeling. Emerging single-cell, spatial, proteomic, and metabolomic approaches may help define fibrosis-associated molecular phenotypes, support patient stratification, and guide future mechanism-informed therapeutic strategies. The image represents a conceptual synthesis of published evidence; solid arrows indicate relationships supported by available OA evidence, whereas dashed arrows indicate more tentative relationships or emerging concepts supported predominantly by cross-disease evidence.

## 2 Cellular reprogramming drives fibrotic remodeling in OA

Fibrotic remodeling in OA involves cellular-state changes within the joint microenvironment in addition to ECM accumulation (6, 10, 12). Among the diverse stromal and immune populations, FLS represent important stromal effectors that integrate inflammatory, biochemical, and mechanical cues during fibrotic remodeling (8, 11, 13). However, stromal reprogramming should be regarded as a dynamic and heterogeneous process involving multiple stromal cell states rather than a single fibroblast phenotype, and may extend beyond conventional fibroblast activation or FMT.

### 2.1 Fibroblast-to-myofibroblast transition as a key cellular process

FMT is an important feature of OA-associated fibrotic remodeling, during which resident fibroblasts acquire a contractile and matrix-producing phenotype (6, 11, 12). Myofibroblasts are characterized by the expression of α-smooth muscle actin (α-SMA), enhanced cytoskeletal organization, increased contractility, excessive production of fibrillar collagens and fibronectin, and localization within fibrotically remodeled tissues (23). In OA synovium, this transition appears to persist rather than resolve as in physiological wound healing, contributing to progressive ECM accumulation and tissue stiffening (6, 8, 12, 23).

Importantly, FMT in OA should be viewed as a continuum of cellular states rather than a binary switch (6). Recent single-cell RNA sequencing (scRNA-seq) studies in human OA tissues and experimental OA models have revealed substantial transcriptional heterogeneity among synovial and infrapatellar fat-pad fibroblasts (24–26). Specific findings include fibroblast populations associated with chondrocyte injury and profibrotic remodeling in human OA tissues (24, 25), as well as RSPO2-expressing lining fibroblasts implicated in pathological cell–cell crosstalk in a murine post-traumatic OA model (26). Related studies in rheumatoid arthritis have provided important methodological and conceptual insights into fibroblast-state diversity, but should not be regarded as direct evidence of OA fibrosis (27–29). However, these transcriptionally defined clusters should not be interpreted as stable cell lineages. Current single-cell datasets are largely cross-sectional, and trajectory inference, pseudotime reconstruction, or RNA velocity analyses infer potential state transitions rather than directly demonstrating lineage relationships (26). Collectively, these findings suggest that fibroblast heterogeneity likely reflects a combination of resident stromal populations, activation-dependent cellular states, and transitional differentiation programs, although their long-term stability and functional significance require further investigation (24–28).

### 2.2 Stromal–immune crosstalk and the fibrotic niche

Fibrotic remodeling in OA is not a fibroblast-autonomous process but arises from dynamic interactions between stromal and immune compartments (3, 30, 31). Synovial macrophages, including tissue-resident and recruited populations, play important roles in shaping the inflammatory and potentially profibrotic microenvironment by producing cytokines such as IL-1β, TNF-α, and IL-6 (3, 32, 33). These signals amplify inflammation and may promote fibroblast activation and profibrotic responses (15, 32). In turn, activated fibroblasts can shape the immune microenvironment through the secretion of chemokines, cytokines, extracellular vesicles, and matrix-derived signals that regulate immune-cell recruitment, retention, and activation (3, 30). This bidirectional crosstalk may reinforce a self-sustaining inflammatory–fibrotic niche (3, 15).

Beyond macrophages, additional innate and adaptive immune populations may contribute to stromal-cell activation and broader joint remodeling (34). Single-cell studies in post-traumatic OA models have identified neutrophils, monocytes, B cells, T cells, natural killer cells, and dendritic cells, together with transcriptionally distinct tissue-resident and recruited macrophage populations (33). T-cell subsets, B cells, dendritic cells, mast cells, and natural killer cells may influence the OA joint microenvironment through cytokine production and cell–cell communication (34). However, direct evidence that these populations regulate persistent stromal activation or fibrotic remodeling in OA remains limited.

In subchondral bone, related immune and remodeling processes also contribute to whole-joint pathology. Recent single-cell profiling of bone marrow lesions (BMLs) identified pro-inflammatory and senescence-associated monocyte states and aberrant BML–cartilage crosstalk associated with cartilage damage in OA (35). These findings support a role for the subchondral immune microenvironment in OA progression but should not be interpreted as direct evidence that BMLs, subchondral sclerosis, and fibrosis are equivalent pathological processes.

ECM remodeling may further reinforce inflammatory–stromal communication by generating matrix-derived damage-associated molecular patterns (DAMPs) (34). ECM-derived DAMPs and matrix degradation products have been implicated in activating pattern-recognition receptors, including Toll-like receptors, and amplifying innate immune signaling in OA (34). Soluble biglycan represents one experimentally validated example and has been shown to activate TLR4–NF-κB signaling in OA chondrocytes and cartilage explants (36). Activation of this pathway promotes inflammatory and catabolic responses, providing a potential mechanism through which ECM damage can amplify local inflammation and further perturb matrix homeostasis (34, 36). This reciprocal interaction may establish a feed-forward damage–inflammation cycle in which ECM disruption enhances innate immune signaling, which in turn promotes further inflammatory and matrix-degrading responses. However, the strength of evidence differs among individual DAMPs, and direct evidence that DAMP–TLR–NF-κB signaling initiates or maintains persistent stromal reprogramming and fibrotic remodeling in OA remains limited.

### 2.3 Cellular plasticity and persistence of fibrotic remodeling

A key unresolved question in OA-associated fibrotic remodeling is why fibrotic responses fail to resolve. Insights from other fibrotic diseases suggest that persistent myofibroblast states may represent an important mechanism of impaired resolution. In fibrotic diseases, persistent myofibroblasts may evade apoptosis or fail to dedifferentiate after the initiating stimulus has subsided (12, 23). This persistence may involve sustained exposure to profibrotic signals, resistance to apoptosis or failure of cellular deactivation, and mechanical reinforcement from a stiffened ECM, while metabolic adaptation may provide additional support for persistent profibrotic states (12, 13, 30, 37). These mechanisms are likely interdependent and collectively contribute to incomplete resolution of tissue repair, although the relative contribution of each process in human OA remains incompletely defined.

Taken together, these findings support a conceptual model in which cellular plasticity is progressively redirected from physiological tissue repair toward persistent stromal reprogramming, thereby stabilizing persistent fibrotic remodeling and promoting chronic joint dysfunction rather than successful tissue resolution (6, 12, 13).

## 3 Molecular networks orchestrating fibrotic remodeling in OA

The transition from adaptive tissue repair to persistent fibrotic remodeling in OA is governed by a tightly interconnected network of signaling pathways that integrate inflammatory, mechanical, metabolic, and stromal regulatory inputs (**Figure 1**) (9, 13). Rather than acting independently, these pathways form a multi-layered regulatory circuit that integrates biochemical and mechanical inputs to sustain fibrotic remodeling (13, 37). Importantly, the strength of evidence supporting individual pathways varies considerably between human OA tissues, experimental OA models, and other fibrotic diseases, and these differences are highlighted throughout this section where appropriate.

### 3.1 TGF-β signaling as a major regulator with context-dependent effects

TGF-β is widely recognized as a major regulator of fibrosis across tissues and plays an important role in OA-associated remodeling (12, 38, 39). In synovial fibroblasts, TGF-β signaling promotes FMT, enhances ECM production, and suppresses matrix degradation through both canonical Smad2/3-dependent signaling and multiple non-canonical signaling cascades, including MAPK, PI3K/Akt, TAK1, and RhoA/ROCK signaling (6, 38).

However, the role of TGF-β in OA is inherently context-dependent (16, 38). While physiological levels of TGF-β are essential for maintaining cartilage homeostasis, dysregulated or excessive signaling may promote pathological remodeling and fibrosis (16, 38). This duality complicates therapeutic targeting, as global inhibition of TGF-β signaling may disrupt cartilage homeostasis and physiological tissue repair (16, 39). This underscores the need for localized or selective modulation of TGF-β signaling. Accordingly, selective modulation of specific downstream pathways rather than global inhibition of TGF-β signaling may provide a more favorable therapeutic strategy.

### 3.2 Inflammation as a permissive and amplifying signal

Chronic low-grade inflammation represents a defining feature of OA and acts as an important permissive and amplifying factor in fibrotic remodeling (3, 32). Pro-inflammatory cytokines—including IL-1β, TNF-α, and IL-6—promote catabolic responses in chondrocytes while simultaneously enhancing fibroblast activation and survival (3, 15).

Notably, inflammatory signaling does not merely coexist with fibrotic remodeling but actively licenses and amplifies fibrotic responses (15, 32). For example, IL-6-mediated activation of the JAK2/STAT3 pathway has been implicated in fibroblast activation and synovial fibrosis in experimental OA (15). Experimental evidence further suggests that inflammatory signaling may interact with TGF-β and mechanotransduction pathways to amplify profibrotic responses, although the precise mechanisms remain incompletely defined. This convergence illustrates that inflammatory cytokines function not only as initiating signals but also as permissive amplifiers that cooperate with profibrotic and mechanical pathways.

### 3.3 Mechanotransduction establishes a feed-forward loop

A characteristic feature of fibrotic tissue remodeling is progressive ECM stiffening, which profoundly alters cellular behavior through mechanotransduction pathways (12, 13). Evidence from experimental OA models and broader fibrosis research suggests that YAP/TAZ act as key mechanosensitive regulators (12, 14). Upstream mechanosensing involves integrin–FAK signaling and RhoA/ROCK-mediated cytoskeletal tension, whereas other mechanosensitive pathways may provide additional routes for translating extracellular forces into cellular responses (37). However, direct evidence demonstrating their role in sustaining persistent stromal reprogramming in human OA remains limited.

In a stiffened microenvironment, YAP/TAZ translocate to the nucleus and drive the expression of genes associated with proliferation, survival, and ECM production (14). Crucially, this activation further enhances fibroblast contractility and matrix deposition, thereby reinforcing ECM remodeling and ECM stiffness (12, 14). Importantly, the mechanical consequences of ECM remodeling depend not only on the amount of matrix deposited but also on qualitative changes in matrix composition and organization. Altered collagen organization and crosslinking, together with changes in matrix architecture and viscoelasticity, can modify tissue stiffness, cellular traction forces, and force transmission from the ECM to the cytoskeleton and nucleus (13, 37). These qualitative alterations therefore provide an important mechanistic link between increased matrix production and sustained mechanotransduction.

Based on findings from OA models and broader fibrosis research, this interaction can be conceptualized as a feed-forward loop:

ECM remodeling and stiffening → mechanotransduction/YAP–TAZ activation → increased matrix production and contractility → further ECM remodeling and stiffening (12–14, 37).

Such a loop provides one potential explanation for the persistence of fibrotic remodeling in OA and may contribute to incomplete resolution of tissue remodeling.

### 3.4 Integration of biochemical and mechanical signals

An emerging concept is that fibrotic remodeling in OA is governed by the integration of biochemical signaling (e.g., TGF-β, cytokines) and biomechanical cues (e.g., matrix stiffness) (12, 13). These inputs converge at shared transcriptional regulators and cytoskeletal pathways, enabling cells to interpret complex environmental signals and adopt stable phenotypic states (14, 37). Evidence from OA models and other fibrotic diseases suggests that TGF-β, inflammatory cytokines, YAP/TAZ, PI3K/Akt, and NF-κB pathways may converge on shared transcriptional and cytoskeletal regulators, thereby coordinating biochemical and mechanical responses (10, 14, 39, 40).

This integrative framework suggests that effective therapeutic strategies may require simultaneous targeting of both signaling and mechanical components, rather than focusing on individual pathways in isolation (13).

### 3.5 Metabolic and epigenetic regulation of fibrotic persistence

Beyond inflammatory and mechanical signaling, emerging evidence suggests that metabolic adaptation and epigenetic remodeling contribute to the persistence of fibrotic responses in OA and other fibrotic disorders. Activated fibroblasts and myofibroblasts undergo metabolic changes that support the increased energetic and biosynthetic demands associated with ECM production. Enhanced glycolytic activity, altered mitochondrial function, and reactive oxygen species accumulation have all been implicated in the maintenance of profibrotic cellular states in multiple fibrotic disorders (10, 30, 41), and whether similar metabolic programs govern persistent stromal remodeling in OA remains incompletely understood.

Metabolic adaptation may interact with mechanotransduction pathways. Evidence from broader fibrosis research suggests that matrix stiffness and cytoskeletal tension can influence cellular metabolic programs, although this mechano-metabolic coupling remains poorly defined in OA-associated fibroblasts (30, 40). Such mechano-metabolic interactions may contribute to the persistence of fibroblast activation within the osteoarthritic joint. Multiple metabolic regulators, including nutrient-sensing and hypoxia-responsive pathways, have also been implicated in fibroblast activation in other fibrotic diseases, although their specific roles in OA-associated fibrosis remain incompletely defined (30, 40, 41).

In parallel, epigenetic mechanisms including DNA methylation, histone modifications, and chromatin remodeling may stabilize profibrotic transcriptional programs (10, 12). These changes can maintain fibroblast activation even after the initiating stimulus has diminished, thereby promoting long-term fibrotic remodeling (10, 12, 41). Similarly, most mechanistic evidence linking epigenetic regulation to persistent fibrotic remodeling originates from non-articular fibrotic diseases, with comparatively limited direct evidence available in OA. An emerging concept is that prolonged matrix stiffness may establish a form of mechanical memory through persistent nuclear mechanotransduction and epigenetic stabilization, thereby reinforcing myofibroblast identity even after the initiating mechanical stimulus is reduced (42). However, the concept of mechanical memory is supported primarily by studies in non-articular fibrotic diseases, whereas comparable mechanisms in OA remain largely hypothetical and require further investigation.

Collectively, these findings suggest that metabolic adaptation and epigenetic remodeling may represent reinforcing mechanisms linking inflammatory signaling and mechanotransduction to persistent fibrotic remodeling (10, 12, 41). However, their specific contribution to OA requires further validation.

## 4 Clinical implications and diagnostic challenges

Despite growing recognition of fibrotic remodeling as an important contributor to osteoarthritis (OA) progression, its clinical relevance remains substantially underappreciated (3, 7). Current diagnostic and therapeutic paradigms continue to prioritize cartilage loss and pain management, while fibrotic changes in the synovium and joint capsule, together with pathological remodeling of the subchondral bone, are often overlooked (3, 8).

### 4.1 Fibrotic remodeling as a driver of joint dysfunction

Fibrotic remodeling contributes directly to joint stiffness, reduced range of motion, and persistent pain through capsular thickening and loss of tissue elasticity (7–9). Moreover, a stiffened fibrotic microenvironment may alter local load distribution, contribute to secondary chondrocyte dysfunction, and accelerate cartilage degradation (13, 37). These observations suggest that fibrosis functions as an active contributor to whole-joint pathology rather than merely a late-stage consequence of tissue degeneration. Although arthrofibrosis represents a clinically useful model of persistent fibrosis, its greater inflammatory intensity and more rapid progression distinguish it from typical OA and should be considered when extrapolating mechanistic insights (9, 12, 38).

### 4.2 Current diagnostic challenges and biomarker development

Diagnostic challenges persist because early fibrotic remodeling is difficult to distinguish from inflammation, structural degeneration, and nonspecific joint stiffness (19, 20). Neither conventional radiography nor routine MRI currently provides a validated, fibrosis-specific measure for early synovial or capsular remodeling (7, 20). Histological assessment remains invasive and unsuitable for longitudinal monitoring, and no validated fibrosis-specific biomarker is currently available. These limitations make it difficult to determine the onset, extent, and progression of fibrotic remodeling in individual patients.

To overcome these limitations, considerable effort has been directed toward identifying fibrosis-associated molecular signatures and biomarkers. Emerging analyses of synovial fluid and extracellular vesicles, together with single-cell and spatial transcriptomics, show promise in identifying fibrosis-associated molecular signatures and stromal-cell states (19, 20, 31, 43). For instance, recent scRNA-seq studies have identified synovial and infrapatellar fat-pad fibroblast populations associated with chondrocyte injury, cartilage degeneration, and OA-related tissue remodeling (24, 25). Additionally, single-cell profiling of BMLs—a key feature of subchondral bone pathology—has identified a senescence-associated immune landscape and BML–cartilage signaling interactions implicated in cartilage damage (35). These approaches may facilitate future patient stratification, and provide insights into stromal–immune–chondrocyte interactions associated with whole-joint remodeling (24, 25, 31, 35).

Despite these advances, most candidate markers remain investigational. Many biomarkers lack specificity for fibrotic remodeling and may also reflect inflammation, cartilage degradation, or generalized ECM turnover. Furthermore, their longitudinal reproducibility, tissue origin, validation in independent patient cohorts, and incremental value beyond conventional clinical and imaging measures remain to be established.

### 4.3 Disease heterogeneity and patient stratification

OA is increasingly recognized as a heterogeneous disease characterized by substantial variability in clinical presentation, molecular mechanisms, and therapeutic response (3, 20). Emerging evidence suggests that fibrotic remodeling may contribute differentially across patient populations, with some individuals exhibiting more pronounced stromal activation, ECM accumulation, and synovial remodeling than others (7, 20). Advances in single-cell and spatial-omics approaches have revealed considerable heterogeneity among fibroblast and immune cell populations within the osteoarthritic joint (24, 25, 31, 35). These observations suggest that fibrosis-associated molecular phenotypes may exist, but their biological stability, temporal evolution, and clinical relevance remain unclear. Improved molecular profiling and biomarker-based classification may ultimately facilitate identification of patients with relatively greater stromal reprogramming and fibrotic remodeling (20, 31, 43).

### 4.4 Barriers to clinical translation

Despite substantial progress in elucidating the mechanisms underlying fibrotic remodeling in OA, translation into clinical practice remains limited. Several factors contribute to this gap. First, fibrotic signaling networks are highly interconnected and redundant, reducing the efficacy of interventions targeting single pathways. Second, key mediators such as TGF-β exert context-dependent effects, playing important roles in both tissue repair and pathological remodeling, thereby complicating therapeutic targeting. Third, the lack of validated fibrosis-specific biomarkers hinders early diagnosis, patient stratification, and treatment monitoring (20, 44). Furthermore, the extent and biological significance of fibrotic remodeling are likely to vary across disease stages and patient populations, making it difficult to identify those most likely to benefit from antifibrotic interventions and complicating clinical trial design and interpretation of therapeutic efficacy. Finally, no antifibrotic therapies have yet been approved specifically for OA (17).

Bridging this translational gap will require integrated approaches that combine mechanistic insights with advanced molecular profiling, biomarker development, and carefully designed clinical trials (17, 20, 31). Incorporating fibrosis-associated biomarkers into patient selection strategies may facilitate identification of individuals most likely to benefit from antifibrotic interventions and improve the likelihood of demonstrating therapeutic efficacy. Such biomarker-guided strategies may also improve enrichment of appropriate patient populations in future clinical trials.

## 5 Therapeutic targeting of fibrotic remodeling in osteoarthritis

The growing recognition of fibrosis as an important contributor to osteoarthritis (OA) progression has stimulated the development of therapeutic strategies targeting multiple aspects of the fibrotic process (7, 10, 17). Rather than focusing exclusively on cartilage preservation, emerging approaches aim to modulate fibroblast activation, inflammatory signaling, mechanotransduction, and microenvironmental remodeling (10, 13). Given the complexity and redundancy of profibrotic signaling networks, durable therapeutic benefit will likely require coordinated modulation of multiple pathological pathways rather than inhibition of individual mediators alone (10, 45).

### 5.1 Targeting TGF-β and inflammatory signaling

TGF-β signaling is widely recognized as a central regulator of fibrotic remodeling and therefore represents an attractive therapeutic target (10, 16, 38, 39). Pharmacological inhibition of TGF-β signaling has shown antifibrotic effects in experimental OA and non-articular fibrosis models (38, 39, 46). However, because TGF-β also plays essential roles in tissue repair and cartilage homeostasis, systemic inhibition may result in undesirable effects (16, 39). Clinical translation will therefore require careful consideration of the timing, location, and extent of TGF-β modulation. In particular, the therapeutic window may be stage dependent, as excessive inhibition during adaptive repair could interfere with physiological tissue responses (16, 39). Accordingly, spatially restricted or pathway-selective approaches may offer a strategy to suppress pathological TGF-β activity while preserving its homeostatic functions in cartilage and other joint tissues (16, 39).

Inflammatory pathways provide additional therapeutic opportunities. Cytokines such as IL-1β, TNF-α, and IL-6 contribute to both inflammatory responses within the osteoarthritic joint and may also influence fibrotic remodeling (3, 32). In particular, activation of the IL-6/JAK/STAT pathway has been implicated in fibroblast activation and synovial fibrosis (15). Targeting these pathways may attenuate the inflammatory signals that sustain fibrotic remodeling; however, anti-inflammatory effects should not be assumed to represent direct antifibrotic activity, and their long-term efficacy in preventing progression of fibrotic remodeling remains to be established in OA (3, 17). The pleiotropic and partially redundant functions of inflammatory mediators may further limit the effectiveness of single-cytokine blockade (3, 17, 47). Biomarker-guided patient selection may help identify patients in whom inflammatory signaling is a prominent component of fibrotic remodeling (20, 31), whereas local or rational combination approaches may provide complementary strategies for improving therapeutic specificity and limiting systemic exposure (17, 18).

### 5.2 Targeting mechanobiological pathways

Mechanotransduction has emerged as a promising therapeutic target because fibrotic remodeling is strongly influenced by the physical properties of the tissue microenvironment (13, 37). Progressive ECM stiffening activates mechanosensitive pathways involving integrin–FAK signaling, cytoskeletal tension, and downstream YAP/TAZ activation, thereby promoting fibroblast activation, matrix production, and persistence of the profibrotic fibroblast phenotype (12–14). YAP/TAZ signaling represents a potential therapeutic node for disrupting stiffness-dependent fibroblast activation. However, direct evidence supporting YAP/TAZ-targeted treatment in OA remains limited, and these factors also participate in tissue repair and mechanoadaptation (13, 14). Clinical translation of mechanotransduction-targeted therapies is complicated by the broad physiological functions of YAP/TAZ and related mechanosensitive pathways, raising concerns regarding tissue specificity and off-target effects. Local delivery or targeting of upstream mechanosensing components within fibrotic joint tissues may provide more selective approaches to disrupt pathological mechanical feedback while preserving physiological mechanotransduction (13, 18).

In addition to targeting intracellular mechanotransduction pathways, strategies aimed at modifying ECM architecture or tissue stiffness may help restore a more favorable biomechanical environment (13, 37). Such approaches highlight the importance of considering both biochemical and mechanical determinants of fibrotic remodeling when developing disease-modifying therapies (13). However, therapeutically modifying ECM mechanics without disrupting the structural and load-bearing functions of joint tissues remains a major challenge, emphasizing the need for spatially controlled and tissue-specific interventions (13, 37).

### 5.3 Cell-based and extracellular vesicle-based therapies

Mesenchymal stem cells (MSCs) have attracted considerable attention because of their immunomodulatory, anti-inflammatory, and tissue-reparative properties (48). Rather than functioning primarily through direct tissue replacement, MSCs exert many of their therapeutic effects through paracrine signaling that modulates immune responses, tissue repair, and matrix homeostasis (48).

Despite their therapeutic potential, MSC-based therapies face several challenges, including limited cell survival and retention after administration, donor- and source-dependent variability, difficulties in manufacturing and standardization, and potential safety and regulatory concerns associated with the administration of living cells (48). MSC-derived extracellular vesicles (MSC-EVs) have emerged as a promising cell-free alternative that may retain key paracrine activities of parental MSCs while avoiding some limitations associated with cell-based therapies (49). Compared with cell-based approaches, MSC-EVs may offer practical advantages in storage, handling, and standardization and avoid concerns related to uncontrolled cell persistence or differentiation (49). MSC-EVs carry regulatory proteins, lipids, and microRNAs capable of modulating inflammation, tissue repair, and ECM homeostasis (49). In a preclinical OA model, exosomes derived from miR-140-5p-overexpressing human synovial MSCs enhanced cartilage regeneration and attenuated OA progression in rats (50). However, these findings primarily support chondroprotective and regenerative effects and do not establish a direct effect on OA-associated fibrotic remodeling. Nevertheless, MSC-EV-based therapies face distinct translational challenges, including heterogeneity in vesicle preparations and cargo composition, lack of standardized production and characterization methods, uncertainties regarding dosing and biodistribution, and inefficient targeted delivery (49). Thus, although MSC-EVs offer potential practical advantages over cell-based approaches, their comparative efficacy, safety, and clinical benefits in OA remain to be established.

### 5.4 Antifibrotic drug repurposing

Repurposing approved antifibrotic agents from other diseases represents a practical strategy for accelerating therapeutic development (45). Drugs such as pirfenidone and nintedanib have demonstrated efficacy in pulmonary fibrosis through their ability to slow disease progression and modulate profibrotic processes (51, 52). Although evidence in OA remains limited and largely preclinical, these agents provide proof-of-concept that common profibrotic pathways may represent therapeutically tractable targets across different organs. Whether these findings can be translated to OA remains to be determined (10, 45).

The success of drug repurposing in OA will depend on identifying patient populations most likely to benefit from antifibrotic therapy and establishing biomarkers capable of monitoring treatment response, because fibrosis-associated molecular phenotypes may not be uniformly present across all OA patients (20, 44, 45). Additional preclinical and clinical studies are needed to determine whether these agents can effectively modify OA-associated fibrosis (17). A major challenge is that systemic exposure and treatment-related adverse effects may constrain the long-term use of repurposed antifibrotic agents for a chronic condition such as OA (45, 51, 52). Moreover, efficacy in pulmonary or systemic fibrosis cannot be assumed to translate directly to the distinct cellular and biomechanical environment of the osteoarthritic joint (10, 40, 45). Local delivery and optimized dosing may help limit systemic exposure (18), whereas biomarker-guided selection of patients with prominent fibrosis-associated features may improve the likelihood of therapeutic benefit (20, 44).

### 5.5 Precision medicine approaches

The marked heterogeneity of OA suggests that antifibrotic therapies may not be equally effective in all patients (3, 20). Emerging multi-omics approaches, particularly single-cell and spatial transcriptomics, are beginning to reveal distinct molecular and cellular states associated with disease progression (24, 25, 31, 43). These technologies may facilitate identification of patient subsets characterized by relatively greater stromal activation and fibrotic remodeling, although the stability and clinical relevance of these molecular phenotypes remain to be established (20, 31).

Biomarker-guided patient stratification could improve clinical trial design and enable more personalized therapeutic interventions. Rather than applying antifibrotic therapies uniformly across all patients with OA, precision medicine approaches may enable biomarker-guided selection of patients who are most likely to benefit from therapies targeting fibrosis-associated pathways (20, 31).

However, implementation of such strategies is currently limited by the absence of validated fibrosis-specific biomarkers, uncertainty regarding the stability of fibrosis-associated molecular states over time, and limited external validation across patient cohorts (20, 31, 43). Longitudinal validation and integration of molecular signatures with imaging and clinical phenotypes will therefore be essential before fibrosis-informed stratification can be incorporated into OA trials or routine clinical practice (20, 43).

### 5.6 Systems-level therapeutic strategies

Given the interconnected nature of inflammatory signaling, fibroblast activation, mechanotransduction, and tissue remodeling, targeting individual pathways in isolation may be insufficient to achieve durable therapeutic effects (10, 12, 13, 45). Future interventions may therefore require systems-level strategies that simultaneously modulate multiple components of the fibrotic network (10, 45). Such approaches may help address pathway redundancy and compensatory signaling that can limit the efficacy of single-target interventions (10, 45).

Potential approaches include rational combination therapies, multifunctional biomaterials, and integrated therapeutic regimens designed to modulate complementary components of the fibrotic network (18, 45). As molecular profiling technologies continue to advance, such systems-oriented approaches may provide a framework for developing mechanism-informed and patient-tailored therapeutic strategies (17, 20, 31).

Despite these opportunities, several translational barriers are shared across current antifibrotic strategies, including pathway redundancy, context-dependent signaling, OA heterogeneity, uncertainty regarding optimal treatment timing, limited fibrosis-specific biomarkers, and the absence of validated outcome measures for monitoring fibrotic remodeling (10, 17, 20, 45). Addressing these challenges may therefore require complementary and context-specific strategies rather than increasingly broad pathway inhibition. Potential solutions include local or tissue-selective delivery (18), biomarker-guided patient enrichment and longitudinal monitoring of fibrosis-associated signatures (20, 31), and rational combination therapies and trial designs that account for disease stage and molecular phenotype (17, 45). Together, these approaches may provide a more realistic path toward disease-modifying interventions that disrupt pathological fibrotic remodeling while preserving physiological tissue repair.

## 6 Discussion and perspective

Fibrotic remodeling is increasingly recognized as an important component of osteoarthritis (OA) progression, linking chronic inflammation, stromal cell activation, and biomechanical alterations within the joint microenvironment. Although substantial advances have been made in understanding the molecular and cellular basis of OA-associated fibrosis, several critical questions remain unresolved. Addressing these challenges will be essential for translating mechanistic insights into effective disease-modifying therapies. Importantly, many mechanistic concepts discussed in this review remain incompletely validated in human OA and should therefore be interpreted in light of the currently available evidence.

### 6.1 Reversibility of OA-associated fibrosis

One of the most important unanswered questions is whether OA-associated fibrosis is reversible. In physiological wound healing, myofibroblasts are typically eliminated through apoptosis or return to a quiescent state after tissue repair is completed. In contrast, fibrotic remodeling in OA appears to persist despite the absence of acute injury, suggesting the establishment of self-sustaining pathological programs (10, 12, 42). Direct evidence defining the reversibility of OA-associated fibrotic remodeling is currently limited. Fibrotic remodeling may exhibit stage-dependent plasticity, with early remodeling remaining responsive to intervention, whereas advanced fibrosis may become increasingly resistant due to matrix stiffening, persistent inflammatory signaling, and stabilization of profibrotic cellular states (10, 12, 13, 42). This concept is consistent with the possibility that fibrosis progresses from an initially adaptive repair response toward progressively stabilized pathological remodeling. Determining the temporal window during which fibrotic remodeling remains reversible will have important implications for therapeutic development and clinical trial design (10, 17). Whether therapeutic intervention can restore stromal homeostasis or merely slow further fibrotic remodeling remains an important unresolved question.

Accordingly, persistent stromal reprogramming should currently be regarded as a conceptual framework rather than an established biological state in human OA, pending further longitudinal and functional validation.

### 6.2 Fibrosis-associated OA phenotypes

OA is increasingly recognized as a heterogeneous disease in which the extent of fibrotic remodeling may vary considerably among patients (3, 20). Whether these differences represent stable biological entities, dynamic disease states, or secondary consequences of disease progression remains unknown. Emerging single-cell and spatial omics studies have identified considerable diversity among stromal and immune cell populations within the osteoarthritic joint, suggesting that fibrosis-associated molecular phenotypes may exist (24, 25, 31, 35). However, their biological stability, temporal evolution, and clinical relevance remain to be established. Future studies integrating molecular profiling, imaging, and clinical phenotyping will be required to determine whether these fibrosis-associated signatures can support meaningful patient stratification and guide therapeutic decision-making (20, 31, 44).

### 6.3 Integration of biochemical and mechanical signaling

A central theme emerging from recent studies is the close interaction between biochemical signaling and mechanical regulation during fibrotic remodeling. Pathways such as TGF-β, inflammatory cytokines, and mechanotransduction mediators including YAP/TAZ do not operate independently but rather form interconnected regulatory networks (13–16). Rather than functioning as isolated pathways, these signaling networks exhibit extensive convergence and reciprocal regulation. Progressive ECM stiffening alters cellular responses to biochemical stimuli, while inflammatory and profibrotic signals further modify tissue mechanics through enhanced matrix deposition (13, 14, 37). Understanding how these biochemical and mechanical inputs converge to stabilize pathological cellular states remains an important challenge. Identification of critical regulatory nodes within these integrated networks may provide new opportunities for therapeutic intervention (10, 13). Future studies integrating experimental biology, computational modeling, and quantitative mechanobiology may help identify regulatory nodes that are both biologically important and therapeutically tractable.

### 6.4 Lessons from clinical translation

Despite growing mechanistic understanding of OA-associated fibrotic remodeling, clinical translation remains limited (17, 20). Experience from other fibrotic diseases indicates that clinical development depends on disease-specific patient selection, validated outcome measures, and realistic therapeutic goals, which may involve slowing progression rather than reversing established structural remodeling (45, 53). Several factors may contribute to the limited success of antifibrotic strategies in OA. First, patient heterogeneity may dilute therapeutic effects in clinical trials when biologically distinct populations are analyzed together (20, 31, 44). Second, fibrotic signaling pathways are highly interconnected and redundant, reducing the effectiveness of therapies targeting individual mediators (10, 41). Third, the absence of validated fibrosis-specific biomarkers complicates patient selection and treatment monitoring (19, 20, 44). Finally, many investigational interventions are evaluated after substantial structural remodeling has already developed, which may limit the potential for meaningful reversal (17). These observations suggest that unsuccessful clinical translation may reflect not only limitations of individual therapeutic targets but also insufficient patient stratification and inappropriate timing of intervention. These challenges highlight the need for improved stratification strategies and earlier intervention.

### 6.5 Future directions and emerging opportunities

Rapid advances in scRNA-seq, spatial transcriptomics, proteomics, and computational biology are providing unprecedented opportunities to investigate fibrotic remodeling at high resolution (24, 25, 31, 35). Future studies should focus on identifying fibrosis-associated cellular states, defining molecular signatures linked to disease progression, and establishing biomarkers suitable for clinical application (20, 31, 44). Equally important will be the development of mechanobiology-informed therapeutic approaches capable of targeting both biochemical and physical drivers of fibrosis (13, 37). Integration of multi-omics data, imaging technologies, and appropriately validated artificial intelligence-based analytical frameworks may facilitate patient stratification and improve prediction of therapeutic responses (31, 44, 54). Such efforts have the potential to accelerate the development of precision medicine strategies for OA, similar to biomarker-guided therapeutic development in several other fibrotic diseases (17, 53). Equally important will be rigorous validation of candidate biomarkers and molecular signatures in independent, longitudinal patient cohorts before routine clinical implementation.

## 7 Conclusions

Fibrotic remodeling is increasingly recognized as an important contributor to osteoarthritis progression and whole-joint dysfunction. Current evidence supports a conceptual model in which persistent stromal reprogramming, inflammatory signaling, mechanobiological adaptation, and ECM remodeling interact to reinforce tissue stiffening and impaired resolution within the osteoarthritic joint. Rather than representing an isolated pathological process, fibrotic remodeling may be viewed as an emergent systems-level consequence of persistent stromal reprogramming, a conceptual framework that requires further validation in human OA.

Despite substantial mechanistic advances, important questions remain regarding the reversibility, temporal evolution, and clinical significance of OA-associated fibrosis. Future progress will require integration of mechanistic studies of stromal reprogramming, biomarker development, longitudinal molecular profiling, and carefully designed clinical studies to determine when, in whom, and to what extent fibrosis-directed interventions may provide therapeutic benefit. Future studies should determine whether persistent stromal reprogramming occurs across different joint compartments and whether shared or tissue-specific mechanisms underlie whole-joint fibrotic remodeling. Ultimately, the clinical relevance of this framework will depend on demonstrating that targeting fibrotic remodeling improves meaningful patient outcomes beyond current symptom-oriented therapies.
